# Sequential intraventricular injection of tigecycline and polymyxin B in the treatment of intracranial *Acinetobacter baumannii* infection after trauma: a case report and review of the literature

**DOI:** 10.1186/s40779-020-00253-9

**Published:** 2020-05-10

**Authors:** Li Zhong, Xue-Zhi Shi, Lei Su, Zhi-Feng Liu

**Affiliations:** 1 Second Clinical College of Guangzhou University of Chinese Medicine, Guangzhou, 510515 China; 2 Department of Critical Care Medicine, the First Affiliated Hospital of Guizhou University of Chinese Medicine, Guiyang, 550001 China; 3 Department of Critical Care Medicine, General Hospital of Southern Theater Command of PLA, Guangzhou, 510010 China; 4 Key Laboratory of Hot Zone Trauma Care and Tissue Repair of PLA, General Hospital of Southern Theater Command of PLA, Guangzhou, 510010 China

**Keywords:** Intraventricular injection of tigecycline, Polymyxin B, Intracranial infection, *Acinetobacter baumannii*

## Abstract

**Background:**

Intracranial infection after craniotomy is one of the most serious postoperative complications, especially multidrug-resistant (MDR) or extensively drug-resistant (XDR) bacterial meningitis, and strongly affects the prognosis of patients. Current treatment experience regarding these infections is scarce.

**Case presentation:**

We report a case of severe intracranial infection of XDR *Acinetobacter baumannii (A. baumannii)* that was treated by intravenous (IV) injection, sequential intraventricular (IVT) injection of tigecycline and polymyxin B, and other anti-infective drugs. Good results were obtained, and the patient was eventually discharged from the hospital. This case is characterized by intracranial infection.

**Conclusions:**

The polymyxin B IV + IVT pathway is an ideal treatment strategy for XDR *A. baumannii*. The tigecycline IVT pathway is also a safe treatment option.

## Background

Intracranial infection after craniotomy is one of the most serious postoperative complications [1]. In two studies, the incidence of bacterial intracranial infections after neurosurgery was 0.3 and 1.5%, respectively [2, 3]. In the United States, a study conducted between 1998 and 2007 showed that the mortality of postoperative intracranial infection was 14.8% [4]. Trauma, neurosurgery, cerebrospinal fluid (CSF) leakage, and external devices are all risk factors for bacteria to enter the CSF [3]. The bacteria causing intracranial infection are mainly gram-negative and gram-positive bacteria. In recent years, the proportion of intracranial infections caused by *Acinetobacter baumannii (A. baumanni)* has been increasing, and the incidence of hospital bacterial meningitis caused by *A. baumannii* accounts for 3.6 to 11.2% of cases [5]. In another study showing a higher proportion of *A. baumannii* meningitis from January 2004 to December 2015, in 134 patients with bacterial meningitis, gram-negative bacteria accounted for 58.2%, gram-positive bacteria accounted for 41.8%, and the most common microorganisms were *A. baumannii*, accounting for 34.8%; the comparison of mortality data showed that mortality among patients with gram-negative bacteria was greater than that among patients with gram-positive bacteria [6]. A South Korean study showed that *A. baumannii* accounted for 32.5% of the pathogens studied in 91 patients with bacterial meningitis with an overall mortality of 16.5% in 91 patients and 26.9% in those with *Acinetobacter* infection [7]. Overall, *A. baumannii* deaths worldwide range from 15.0 to 71.5% [8, 9]. In the past several decades, the drug resistance rate of *A. baumannii* has also increased significantly. A microbial monitoring experiment reported that the drug resistance rate of *A. baumannii* was 30% [10]. In the face of such high mortality and drug resistance rates, the treatment of intracranial infection caused by *A. baumannii* is urgently necessary. Because the permeability of the blood-brain barrier is poor and bacterial drug resistance increases, the sensitive bacterial inhibition concentration (MIC) increases significantly, and different antibiotics have different abilities to enter the CSF, limiting the treatment options for *Acinetobacter* intracranial infection; therefore, the use of only intravenous (IV) anti-infection drugs cannot effectively treat intracranial infection. Recent research has combined IV drug use and intraventricular (IVT) medication management to enhance drug concentrations at the site of infection [11, 12]. Currently, the IVT administration of drugs for gram-negative bacterial meningitis includes aminoglycosides, colistin and polymyxin B. According to recent reports on the treatment of central nervous system (CNS) infection, the effective treatment for multidrug-resistant (MDR) or extensively drug-resistant (XDR) *A. baumannii* infection is the combination of IV and IVT drugs, and the drugs used for this treatment are mainly polymyxin B and tigecycline [11, 13, 14]. However, the relevant reports are all studies with small sample sizes or are case reports. We report a case of severe craniocerebral trauma complicated by intracranial infection of XDR *A. baumannii* in the intensive care unit. The treatment process of sequential intrathecal injection of tigecycline and polymyxin B was successful, and the experience is shared in this report.

## Case presentation

**Fig. 1:**
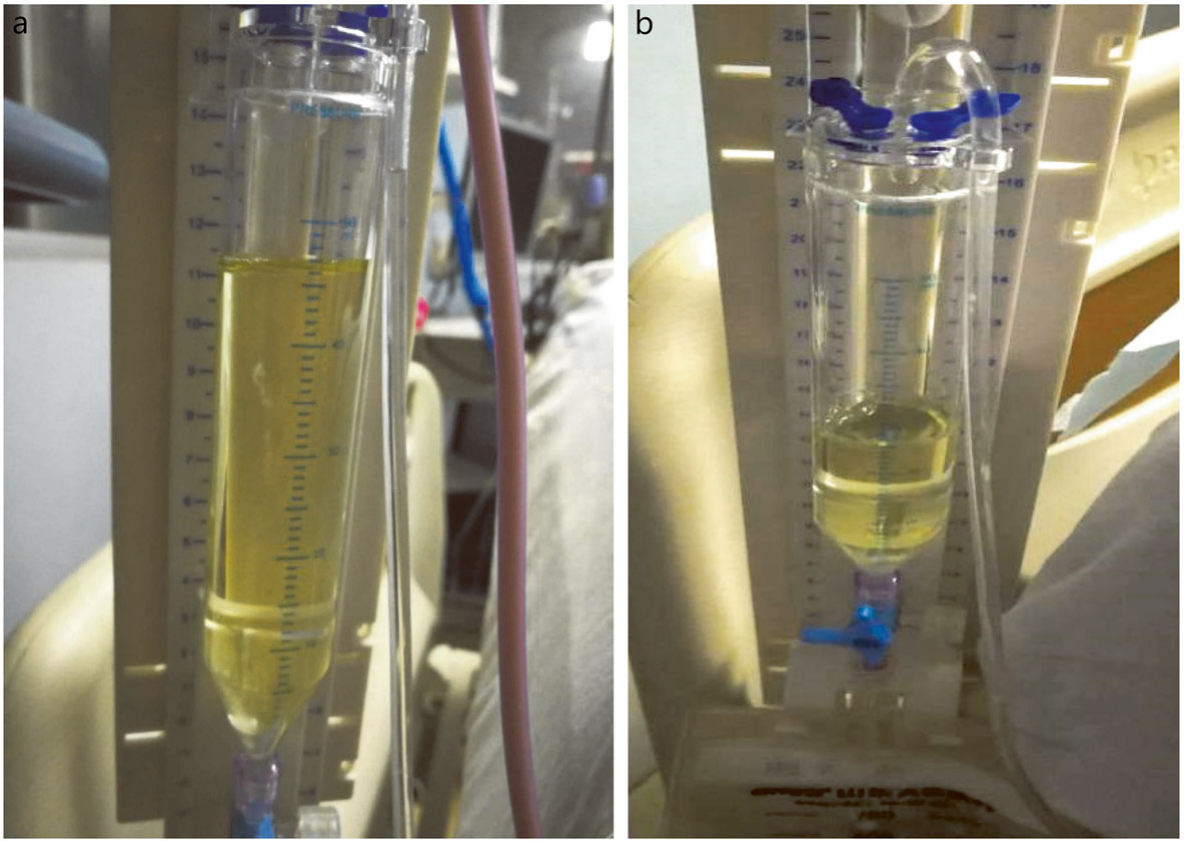
Changes in cerebrospinal fluid (CSF) in patient with intracranial *A. baumanni* infection before and after treatment. a: CSF on July 8; b: CSF on August 2

**Fig. 2:**
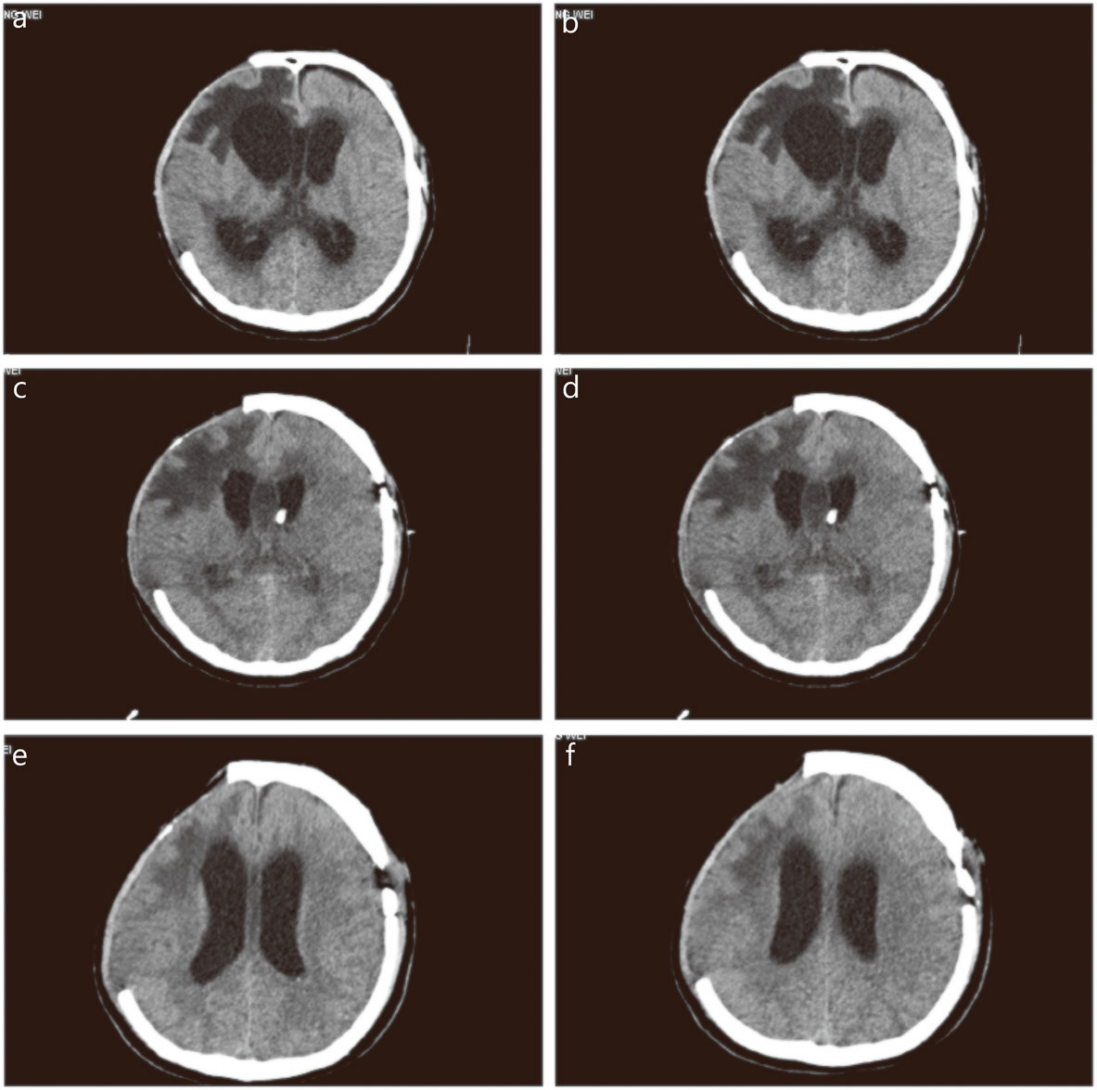
Brain CT after admission in patient with intracranial *A. baumanni* infection. a. Before the treatment on July 3, large low-density shadows were seen in the right frontotemporal parietal lobe; b. Before the treatment on July 3, large low-density shadows appeared in the bilateral lateral ventricles and the third ventricle dilated; c. During the treatment on July 16, bilateral lateral ventricle and the third ventricle were dilated, and hydrocephalus was better than before; d. During the treatment on July 16, a free tube shadow was seen through the left frontal bone to the anterior horn of the left ventricle; e. After treatment on August 2, the original free tube shadow was removed; f. After treatment on August 2, bilateral lateral ventricular hydrocephalus was observed

**Fig. 3:**
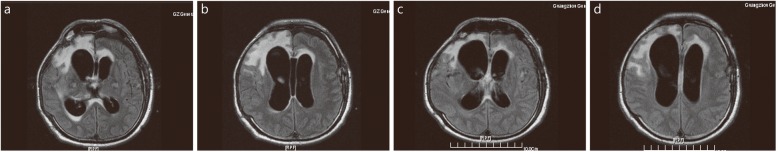
Brain MRI in patient with intracranial *A. baumanni* infection before and after treatment. a.: On July 4, subarachnoid hemorrhage occurred in the right frontal lobe; b. On July 4, midline, subdural effusion and hydrocephalus occurred in the left frontal part; c. On July 24, subarachnoid hemorrhage in the right frontal lobe; d. On July 24, midline and subdural effusion in the left frontal part were better than before

The patient, a 33-year-old male, was treated with intracranial hematoma clearance and bone flap decompression due to severe craniocerebral trauma caused by a high fall four months earlier. Twenty days after the surgery, consciousness disturbance was aggravated. Head computed tomography (CT) examination indicated increased hydrocephalus and CSF leakage, and ventricular borehole drainage and lumbar cistern drainage were performed several times (June 12, 2018). The CSF leukocyte count increased to 4000.0 × 10^6^/L (Table 1). *Staphylococcus aureus* was found in the CSF culture, and the infection was treated with meropenem + vancomycin IV combined with vancomycin IVT. By July 2, 2018, the white blood cell count was reduced to 1.9 × 10^6^/L, blood glucose was 3.4 mmol/L, and protein was 0.30 g/L. Brain CT and magnetic resonance imaging (MRI) reexamination still indicated severe hydrocephalus (Fig. 2a and b, Fig. 3a and b), and lumbar cistern drainage was performed again (July 8, 2018). The patients’ bone window pressure at night increased significantly; the CSF exhibited a deep yellow color with turbidity and flocculation (Fig. 1a); and the patient had a high fever, with the highest temperature being 39.5 °C. The CSF white blood cell count was 29,887.0 × 10^6^/L, the red blood cell count was 127 × 10^6^/L, blood glucose was 0.1 mmol/L, protein was 0.95 g/L, and CSF cultures indicated XDR *A. baumannii* (Table 2). Left ventricular borehole drainage was performed (with holes closed for 1 h before opening), and the antibiotics were adjusted to meropenem 2 g × q8 h + vancomycin 1 g × q12 h + tigecycline 100 mg × q12 h IV and tigecycline 5 mg × q12 h IVT. During the 7 days of the use of this regimen, the color of the CSF of the patient gradually faded, and the white blood cells decreased to 790 × 10^6^/L; the blood glucose was 2.7 mmol/L, and the protein was 2.11 g/L (July 15, 2018). The white blood cell count in the CSF was 743 × 10^6^/L. CSF culture indicated that XDR *A. baumannii* was still present, and the indicators of liver function detected were higher than before. Suspected hepatic damage due to tigecycline was not excluded; therefore, we adjusted the antibiotic to be polymyxin B 100 mg × q12 h + meropenem 2 g × q8 h + vancomycin 1 g × q12 h IV anti-infection treatment and gave polymyxin B 10 mg × qd, which was changed to qod × 2w IVT 4 days later. The patient did not have fever after treatment, and the Glasgow Coma Scale (GCS) score increased from E1VTM1 to E1VTM5, which indicated that the patient’s conscious state had improved. Head CT and MRI showed that the effusion was better than the previous absorption (Fig. 2c, d, e, and f, Fig. 3c and d, August 2, 2018). After reexamination, white blood cells in CSF gradually decreased to 31.2 × 10^6^/L, blood glucose was 3.1 mmol/L, and protein was 2.24 g/L. Bacteria were not found in 4 CSF cultures, and the color of the CSF became pale yellow and transparent without flocculation (Fig. 1b, August 3, 2018). The antibiotic was adjusted to ceftazidime 2 g × q8 h + polymyxin B 100 mg × q12 h IV treatment. After further stabilization, the patient was transferred to the hospital for further brain rehabilitation.

**Table 1:** Laboratory examination of cerebrospinal fluid in patient with intracranial *A. baumanni* infection

Time point	WBC (×10^6^/L)	Glu (mmol/L)	Protein (g/L)
June 12, 2018	4000.0	–	–
July 2, 2018	1.9	3.4	0.30
July 8, 2018	29,887.0	0.1	0.95
July 12, 2018	848.0	2.0	1.73
July 14, 2018	790.0	2.7	2.11
July 15, 2018	743.0	2.7	1.77
July 18, 2018	893.0	1.5	1.65
July 21, 2018	144.0	2.2	1.56
July 26, 2018	108.0	3.2	2.59
July 31, 2018	121.0	3.2	1.63
August 2, 2018	31.2	3.1	2.24

**Table 2:** Bacterial culture of CSF in patient with intracranial infection

Antibiotics	MIC	Drug sensitivity
Piperacillin and tazobactam	128	R
Ceftazidime	64	R
Cefoperazone/Sulbactam	64	R
Cefepime	32	R
Imipenem	16	R
Meropenem	16	R
Tobramycin	16	R
Minocycline	8	I
Tigecycline	1	S
Colistin	0.5	S
Trimethoprim/Sulfamethoxazole	20	S

MIC: Minimum inhibitory concentration; R: Resistant; I: Intermediate; S: Susceptible

## Discussion

This paper discusses the anti-infection treatment strategy for a case of intracranial infection with XDR *A. baumannii* and describes the effects of tigecycline in its treatment*.*

### Anti-infection treatment strategy of intracranial infection with XDR *A. baumannii*

According to previous reports, the main pathogens of surgical-site infection are gram-positive bacteria, especially *Staphylococcus aureus* [15]. In this case, *Staphylococcus aureus* was found in the CSF culture at the time of the first intracranial infection, which was consistent with the pathogen species mentioned in the report. Vancomycin IV and IVT are the main therapeutic drugs for the intracranial infection of gram-positive cocci [16]. However, in the closed environment of hospitals, the generation of drug-resistant bacteria is becoming increasingly serious. In addition, neurosurgery, CSF leakage, long-term and repeated ventricular drainage, the use of broad-spectrum antibiotics, long-term mechanical ventilation and stay in the intensive care ward and intracranial hemorrhage are all high-risk factors for the generation of drug-resistant bacteria [17]. The average time to develop *A. baumannii* infection is 12 days (range 40 days) [17]. There have also been reports on the discovery of a previous pathogen, as well as the detection of a new pathogen, and the emergence of drug-resistant bacteria ranges from 3 to 90 days [18]. In this study, the time for the development of *A. baumannii* in CSF culture was 28 days. We know that polymyxin has been in use since the 1950s, but its use rate has decreased year by year due to severe nephrotoxicity and neurotoxicity. However, in recent years, researchers have found that polymyxin is active against both MDR and XDR gram-negative bacteria, including *A. baumannii*, and researchers have attempted to use the IVT pathway in patients with intracranial infection [5, 13, 19]. Colistin has CNS permeability. In recent years, colistin has been recommended in studies to treat intracranial infection caused by multidrug-resistant bacteria. The IVT and IV injection of colistin are currently also options for treatment, and some success has been achieved in the cases studied [5, 7]. In this report, *A. baumannii* was found in the CSF culture when the white blood cell counts in the CSF had become elevated again in the patient. The drug sensitivity results indicated that the patient was sensitive to polymyxin B. At that time, there was no polymyxin B or polymyxin E in the Chinese market. After the patient’s wife purchased polymyxin B from Hong Kong, we gave the patient this drug. Therefore, polymyxin B IV and IVT schemes, namely, polymyxin B 100 mg × q12 h IV and 10 mg × qd, were used and changed to qod × 2w IVT 4 days later. According to the guidelines issued by the American Academy of Infectious Diseases for the treatment of bacterial meningitis, the dose of ventricular colistin should be 10 mg per day [20] with satisfactory results. During this process, the renal function of the patient was closely monitored, and no nephrotoxicity or neurotoxic side effects were observed. For polymyxin B injection, based on the evidence provided and the IV and IVT joint scheme, colistin is an ideal drug because it has good antibacterial activity against MDR or XDR *A. baumannii*, but clinicians need to be mindful of mucin-related complications, including chemical meningitis inflammation, chemical ventricle inflammation, seizures and horsetail nerve syndrome (there have been reported incidences of 21.7%) [5]. Close monitoring of renal function during treatment, more careful management of fluids and electrolytes, and the avoidance of combination with other drugs known to be nephrotoxic may reduce the incidence of associated side effects.

### Characteristics of tigecycline in the treatment of intracranial XDR *A. baumannii*

According to recent progress in the treatment of CNS infection, tigecycline is often the first clinical application. Because of the ammonia acyl ring element compounds of this drug, tigecycline has a wide variety of activities among MDR pathogens. Tigecycline usually displays good antibacterial activity against resistant gram-negative and gram-positive bacteria, as do many antimicrobial drugs according to synergy, but due to the lack of penetration to the CNS, their serum concentration in the CSF is only 11% [21, 22]. Therefore, the IV injection of tigecycline has no significant effect on patients with intracranial infection, and routine intravenous use of tigecycline is not recommended for *A. baumannii* meningitis. In recent years, there have been notably few reports of the successful IVT use of tigecycline [14, 23–25]. In this report, when *A. baumannii* was found in the first culture, we used tigecycline 100 mg × q12 h IV and 5 mg × q12 h IVT according to the drug sensitivity. During the 7 days of use, the number of white blood cells in the CSF repeatedly decreased, and the clinical symptoms of the patient were significantly improved without neurotoxic side effects. We have no pharmacokinetic data in our report because our hospital currently cannot monitor the concentrations of tigecycline or polymyxin B in serum and CSF. However, according to the literature, various dosages of tigecycline (49 mg intravenously and 1 mg intraventricularly every 12 h, 45 mg intravenously and 5 mg intraventricularly every 12 h, or 40 mg intravenously and 10 mg intraventricularly every 12 h) are well tolerated and effective. After a dose of 5 mg intraventricularly every 12 h, CSF peak-trough concentrations were approximately (327.0–1.7 mg/L). The dose of tigecycline we used was consistent with the reported dose in the literature; therefore, the corresponding pharmacokinetics should be consistent. Moreover, according to the guidelines issued by the American Academy of Infectious Diseases for the treatment of bacterial meningitis, the dose of ventricular colistin should be 10 mg/d, but there is a lack of information on the pharmacokinetics of polymyxin B after administration to patients via routes other than IV injection (e.g., inhalation, intrathecal injection, and IVT injection). The literature reported similar antibacterial activity in vitro, and two studies have reported the pharmacokinetics (PK) results of colistin after intrathecal administration (ITH)/IVT colistin methanesulfonate (CMS) application in patients. In one of the studies, when patients were treated with CMS 5.22 mg/d, the CSF concentrations of colistin were continuously above 2 μg/ml, and the C_max_/MIC ratio was ≥3.5.

## Conclusion

IVT tigecycline injection seems to be a safe treatment for *A. baumannii* intracranial infection. Unfortunately, we did not measure tigecycline concentration in CSF. Currently, there are no large-sample clinical RCT studies, and only scarce reports have been presented, but the effectiveness of the IVT injection of tigecycline still needs to be further studied.

## Data Availability

Not applicable.

## Abbreviations

CNS: Central nervous system
CSF: Cerebrospinal fluid
CT: Computed tomography
GCS: Glasgow Coma Scale
IV: Intravenous
IVT: Intraventricular
ITH: Intrathecal administration
MIC: Minimum inhibitory concentration
MRI: Magnetic resonance imaging
MDR: Mulitidrug-resistant
PK: Pharmacokinetics
XDR: Extensively drug-resistance
